# Validating Grading of Aesthetic Outcomes of Web Space Reconstruction for Finger Syndactyly: Crowdsourcing Public Perceptions Using Amazon Mechanical Turk

**DOI:** 10.1093/asjof/ojaa046

**Published:** 2020-11-07

**Authors:** Cory K Mayfield, Ian Thomas, Orr Shauly, Daniel J Gould, Mitchel Seruya

**Affiliations:** Department of Plastic and Reconstructive Surgery, Keck School of Medicine, University of Southern California, Los Angeles, CA, USA; Department of Plastic and Reconstructive Surgery, Keck School of Medicine, University of Southern California, Los Angeles, CA, USA; Department of Plastic and Reconstructive Surgery, Keck School of Medicine, University of Southern California, Los Angeles, CA, USA; Department of Plastic and Reconstructive Surgery, Keck School of Medicine, University of Southern California, Los Angeles, CA, USA; Department of Plastic and Reconstructive Surgery, Keck School of Medicine, University of Southern California, Los Angeles, CA, USA

## Abstract

**Background:**

It has recently been attempted in the literature to analyze the aesthetic outcomes of syndactyly web space reconstruction utilizing dorsal pentagonal advancement flaps and dorsal rectangular flaps with skin grafting. The study utilized a categorical grading system for evaluating the aesthetic outcomes of reconstruction to be used in conjunction with a visual analog scale (VAS), which has yet to be validated in the assessment of aesthetic outcomes following web space reconstruction.

**Objectives:**

To utilize crowdsourced public perceptions to validate the grading of aesthetic outcomes in web space reconstruction for finger syndactyly.

**Methods:**

A prospective study was conducted of random volunteers recruited through an internet crowdsourcing service to gain responses for a survey to analyze patient opinions toward the aesthetic outcomes of web space reconstruction. Outcomes were graded based on descriptions of the appearance, color, matte, and distortion of the reconstruction.

**Results:**

The excellent dorsal flap demonstrated a mean VAS score of 6.66 (95% confidence interval [CI] = 6.45-6.87), and the very good, good, and poor dorsal flaps had mean VAS scores of 5.94 (95% CI = 5.73-6.15), 4.98 (95% CI = 4.77-5.19), and 3.55 (95% CI = 3.31-3.79), respectively. The odds ratio for receiving an excellent rating was 4.21 (95% CI = 3.04-5.82) for excellent dorsal flap with *P* < 0.0001.

**Conclusions:**

This study confirms and validates the assessment of aesthetic outcomes of web space reconstruction by the Yuan Grading Scale. This evidence may guide future practice such that recommendations can be made to align with the aesthetic preferences of the patient.

Syndactyly is a common congenital malformation that results as a failure of apoptosis and skin recession during early gestation. The true incidence of syndactyly is uncertain with estimates ranging from 1 in 2000 to 1 in 3000 live births.^1^ Web space reconstruction is a key concept in the treatment of syndactyly. Two principles of web space reconstruction are to create cosmetically acceptable and functionally independent digits.^2^ To date, much of the literature on syndactyly has focused on reconstruction and different techniques for surgical management of syndactyly.^2-4^ However, few have utilized objective grading scales to focus on the postoperative aesthetic outcomes.^5-7^

Recently, a study by Yuan et al^7^ attempted to analyze the aesthetic outcomes of syndactyly web space reconstruction utilizing dorsal pentagonal advancement flaps and dorsal rectangular flaps with skin grafting. Within their study, the authors utilized a categorical grading system for evaluating the aesthetic outcomes of reconstruction to be used in conjunction with a visual analog scale (VAS). The grading system utilized was based on the Manchester Scar Scale, which has previously demonstrated interrater reliability, validity, and feasibility for evaluating surgical scars.^8-10^ However, this grading system has yet to be validated in the assessment of aesthetic outcomes following web space reconstruction.

The purpose of the current study was to utilize crowdsourced public perceptions to validate the grading of aesthetic outcomes in web space reconstruction for finger syndactyly. We sought to utilize Amazon Mechanical Turk (MTurk) to acquire a crowdsourced assessment of aesthetic outcomes utilizing global assessment and individual components of the categorical grading system. As there is no current grading system that has been universally utilized in the assessment of aesthetic outcomes following web space reconstruction, we aimed to use objective crowdsourced evaluators to assess validity, reliability, and feasibility of the VAS and a categorical grading system.

## METHODS

A prospective cross-sectional study by survey was conducted of random volunteers recruited through an internet crowdsourcing service (MTurk) between April 3 and April 10, 2019.^11-13^ Several studies have demonstrated that the worker population is extremely representative of the US internet population.^13,14^ This technique has previously been reliably utilized in the plastic surgical literature.^15,16^ Workers are provided with a level of compensation and estimated time of completion and are screened by Amazon for quality responses. We did not allow workers with lower than a 5-star worker rating (the maximum possible score) from participating in the survey. This study was exempt from Institutional Review Board approval, given that this study utilized deidentified survey data, however informed consent was provided by the workers through their contract with Amazon MTurk. 

MTurk workers are required to be above the age of 18 and registered through the Amazon service platform to prevent individuals from multiple survey responses. Surveys were open to 200 people at a time for approximately 24 hours (repeated 5 times), and workers were paid $0.05 per unique response. This allowed us to screen for quality, completeness, and duplicate users before proceeding to collect more data. The survey was created by authors C.K.M. and O.S., with permission provided to include images from the study of Yuan et al^7^ (Supplemental Appendix). The images included in the survey represent the outcomes of both simple and complex web space reconstruction, with a primary focus on the aesthetic qualities (Supplemental Figures 1-4).

### Screening Questions

Although MTurk requires that registered volunteers be above the age of 18, individuals may not be completely truthful when creating their account. To ensure that all surveyed participants were considered adults, the first questions of the survey asked the participants to reenter their age. Any response below the age of 18 immediately disqualified the worker. No other screening questions were administered to maintain a truly diverse representation of the general US population.

### Attention Check Question

To ensure that survey participants were paying close attention to each question and scenario, and to also ensure that the generated data was a valid representation of patient opinions, the following attention check question was included approximately halfway through the survey:

“You opt to undergo a novel surgical procedure that may completely heal your injury with minimal postoperative pain. You will answer 72 exactly to this question regardless of how you feel about this scenario. There is a high chance the surgery will work, but if it does not, you will require much more extensive surgery and will have limited wrist function.” 

Respondents who entered a number anything other than “72” were directed to the end of the survey and were excluded from this study. Those who were excluded were prevented from ever taking this survey again.

### Preference Questions

Crowdsourcing was utilized to gain responses for a survey to analyze patient opinions toward the aesthetic outcomes of web space reconstruction. Outcomes were graded based on the findings from Yuan et al^7^ and included excellent, very good, good, and poor. These outcomes were judged based on 4 categories including a description of the appearance, color, matte, and distortion. Included in the survey conducted was an overall utility score (vertical VAS scale) ranging from 0 to 100 with 0 representing an indistinguishable finger and 100 representing a perfect reconstruction. These utility scores represented interval survey data and were then analyzed across both treatment options and all patient-reported demographics.

### Data Analysis

Data from the survey were pooled and assessed using Microsoft Excel 2016 (Redmond, WA). Statistics were performed using Stata (College Station, TX) with continuous data evaluated using 2-tailed 2-sample unequal variances *t*-tests (significance at alpha = 0.05).

## RESULTS

### Patient Characteristics

A total of 590 MTurk participants were interested in the survey. However, 150 (25%) of these were excluded due to either failing to meet inclusion criteria (1) or failing to fully complete the survey (149). Therefore, the 440 participants who met the inclusion criteria (properly answered screening and attention check questions) and completed the survey were included in this study. This screening methodology ensures that data are derived from those participants who were fully attentive to the survey materials.

### Study Demographics

The demographics of participants in this survey can be found in Table 1. The majority of our survey participants were between the ages of 25 and 34 (56.6%) with 83% of participants between the ages of 18 and 44. Females and males comprised of 55% and 45% of participants, respectively, indicating both sexes were roughly equally represented in this study. By race, the majority of participants were White (56%) followed by Asian (22.5%). Nonwhite Hispanics, African Americans, American Indians, and Pacific Islanders made up the remaining 21% of participants. Finally, each annual income cohort was appropriately represented with at least 10% of participants in each income cohort except for the >$100,000 cohort (7%).

**Table 1.** Demographics of All Study Participants Who Were Eligible and Completed the Survey (N = 440)

	No. of Participants (%)
Age	
18-24	57 (13%)
25-34	249 (57%)
35-44	59 (13%)
45-54	39 (9%)
55-64	27 (6%)
>65	9 (2%)
Sex	
Male	200 (45%)
Female	240 (55%)
Race	
White	247 (56%)
Asian	99 (22.5%)
Nonwhite Hispanic	19 (4%)
African American	37 (8%)
American Indian	33 (8%)
Pacific Islander	5 (1%)
Annual income	
<$50,000	198 (45%)
$50,000-$74,999	150 (34%)
$75,000-$100,000	60 (14%)
>$100,000	32 (7%)

With respect to the general population, the exact Fisher’s test was performed in order to compare the study population to the 2019 national consensus data in the United States. It was found that the age distribution of this study (Fisher’s exact test value = 0.59) and gender distribution (Fisher’s exact test value = 0.67) were not significantly different than that of the general US population at a *P* < 0.05. However, the race demographic information was statistically significantly different than that of the predominantly Caucasian general US population (Fisher’s exact test value = 0.004). Furthermore, 2018 national consensus data demonstrated a significantly larger percentage of families with an annual household income of greater than $100,000 (38.4%, Fisher’s exact test value < 0.00001).

### Aesthetic Outcomes

**Figure 1.**
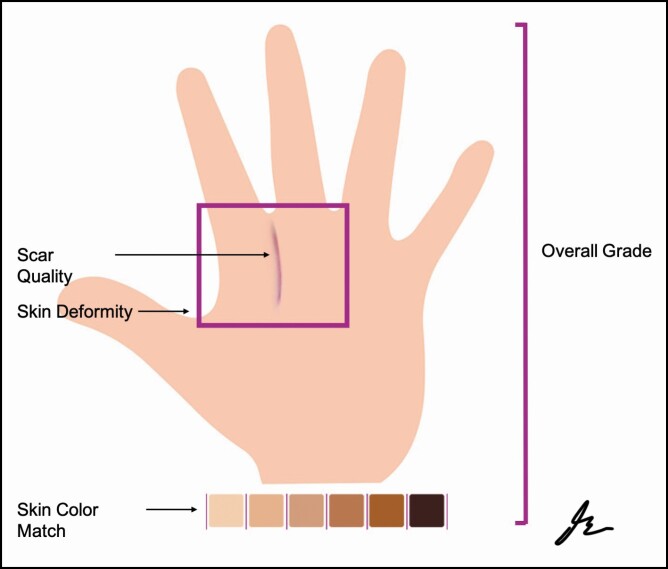
Schematic of categorical criteria evaluated by the grading system. Participants were asked to evaluate an overall grade, scar quality, skin deformity, and skin color match.

Study participants were asked to evaluate 4 dorsal reconstruction flaps predesignated as excellent, very good, good, or poor for the overall grade and various aesthetic criteria (Figure 1). The participants were blinded to the predesignated grade assigned to each flap. Table 2 illustrates the categorical criteria included in the survey. For overall grade, each participant was asked “On a scale of 0–10, where 0 represents an indistinguishable finger (does not look like a finger at all), and 10 represents a perfect looking finger/hand, how would you rate the overall appearance of this child’s fingers/hand?” For overall grade, participants were asked “Using the following criteria, and based on your answers to the above questions, please provide an overall grade to this reconstructed finger. Excellent = Equal appearance to surrounding skin in color, matte, and no skin distortion; Very Good = Similar appearance to surrounding skin with mild skin distortion; Good = Shiny appearance compared to surrounding skin with moderate skin distortion; Poor = Obvious scare with severe skin distortion.” For scar quality, participants were asked “Please note if the skin overlying the fingers and/or scar is ‘matte’ (not shiny) or ‘shiny.’” For skin color match, participants were asked “Please describe the skin color of the reconstructed fingers compared to the skin color of the rest of the child’s hand.” For skin deformity, participants were asked “Please describe how distorted the reconstructed hand/fingers look compared to what you believe a normal hand/fingers should look like.”

**Table 2.** Categorical Grading System Utilized by Yuan et al

Grade	Description	Color	Matte	Distortion
Excellent (E)	Equal appearance to surrounding skin in color, matte, and there is no skin distortion present.	Perfect match	Matte	None
Very Good (VG)	Similar appearance to surrounding skin with mild skin distortion.	Slight mismatch	Matte	Mild
Good (G)	Shiny appearance compared to the surrounding skin with moderate skin distortion.	Obvious mismatch	Shiny	Moderate
Poor (P)	Obvious scar visible with severe skin distortion.	Gross mismatch	Shiny	Severe

**Figure 2.**
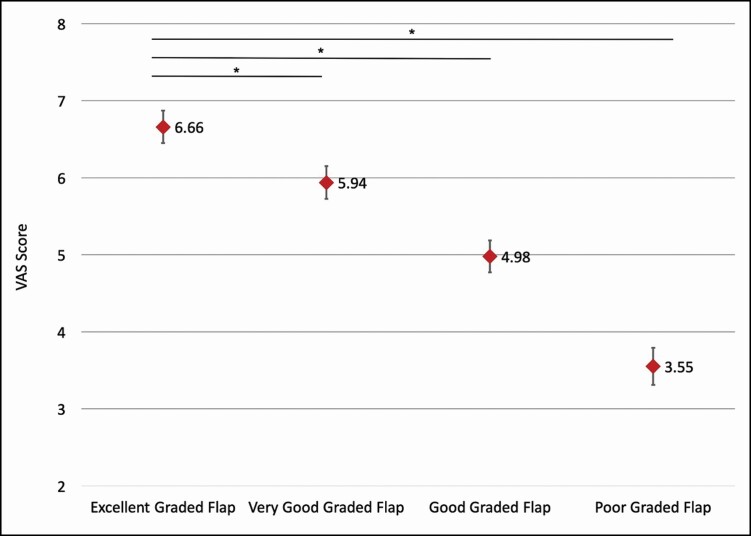
Mean visual analog scale (VAS) scores reported with 95% confidence intervals. Mean VAS scores with 95% confidence intervals are shown for each repair used in the survey. One-way *t*-tests were performed for the difference in the mean between the dorsal rectangular flap and each pentagonal advancement flap (*P < 0.001).

Overall, the participants gave the excellent dorsal flap a mean VAS score of 6.66 with a 95% confidence interval (CI) of 6.45–6.87 (Figure 2). Meanwhile the very good, good, and poor dorsal flaps were given mean VAS scores of 5.94 (95% CI = 5.73-6.15), 4.98 (95% CI = 4.77-5.19), and 3.55 (95% CI = 3.31-3.79), respectively. One-way *t*-test for difference of the means between the excellent flap and each of the very good, good, and poor flaps revealed *P* < 0.0001 in each case. Additionally, single-factor ANOVA analysis of the VAS scores for the 4 reconstructive flaps resulted in a *P*-value < 0.0001.

The analysis of VAS scores based on age, sex, race, and annual income is summarized in Table 3. Males and females gave the excellent dorsal flap VAS scores of 6.38 (95% CI = 6.07-6.70) and 6.89 (95% CI = 6.61-7.17) as compared with 5.75 (95% CI = 5.44-6.05) and 6.10 (95% CI = 5.81-6.39) to the very good dorsal flap. The 18–24 age range gave a higher VAS score to the excellent dorsal flap than the very good dorsal flap, 6.53 (95% CI = 6.26-6.80) as compared with 5.78 (95% CI =5.50-6.06). All other age cohorts gave a higher VAS score to the excellent dorsal flap than to the good dorsal flap. White participants were the only race that gave a higher VAS score to the excellent dorsal flap (6.99 with 95% CI = 6.71-7.27) than the very good dorsal flap (6.17 with 95% CI = 5.89-6.45). Lastly, the $50,000–$74,999 income range also gave a higher VAS score to the excellent dorsal flap than the very good dorsal flap.

**Table 3.** Mean Visual Analog Scale Scores for the “Very Good,” “Good,” and “Poor” Dorsal Pentagonal Flap Images Provided to Study Participants

	Dorsal Rectangular Flap Mean VAS (95% CI)	“Very Good” Dorsal Pentagonal Flap Mean VAS (95% CI)	“Good” Dorsal Pentagonal Flap Mean VAS (95% CI)	“Poor” Dorsal Pentagonal Flap Mean VAS (95% CI)
Total	6.66 (6.45-6.87)^a^	5.94 (5.73-6.15)	4.98 (4.77-5.19)	3.55 (3.31-3.79)
Age				
18-24	6.45 (5.87-7.04)^a^	5.45 (4.91-6.09)	4.67 (4.13-5.21)	3.51 (2.91-4.11)
25-34	6.53 (6.26-6.80)^a^	5.78 (5.50-6.06)	5.07 (4.79-5.35)	3.93 (3.60-4.26)
35-44	6.45 (5.80-7.09)^a^	5.69 (5.11-6.27)	4.75 (4.15-5.35)	2.80 (2.17-3.44)
45-54	7.36 (6.73-7.98)^a^	6.98 (6.29-7.66)	5.24 (4.56-5.92)	3.17 (2.46-3.89)
55-64	7.62 (6.90-8.34)^a^	7.20 (6.35-8.05)	5.01 (4.14-5.89)	2.57 (1.70-3.44)
>65	7.11 (5.79-8.44)^a^	6.48 (5.03-7.93)	4.62 (3.55-5.70)	2.86 (1.72-3.99)
Sex				
Male	6.38 (6.07-6.70)^a^	5.75 (5.44-6.05)	4.80 (4.50-5.11)	3.51 (3.16-3.86)
Female	6.89 (6.61-7.17)^a^	6.10 (5.81-6.39)	5.12 (4.85-5.40)	3.59 (3.26-3.92)
Race				
White	6.99 (6.71-7.27)^a^	6.17 (5.89-6.45)	4.88 (4.62-5.15)	3.11 (2.82-3.40)
Asian	6.07 (5.57-6.57)^a^	5.63 (5.14-6.12)	5.11 (4.62-5.59)	4.23 (3.68-4.78)
Nonwhite Hispanic	6.98 (6.01-7.98)^a^	5.79 (4.58-7.01)	4.59 (3.46-5.73)	3.28 (2.16-4.39)
African American	6.42 (5.75-7.10)^a^	5.41 (4.67-6.15)	4.99 (4.29-5.70)	3.10 (2.23-3.97)
American Indian	6.07 (5.49-6.66)^a^	5.87 (5.20-6.53)	5.35 (4.73-5.98)	5.24 (4.46-6.01)
Pacific Islander	6.46 (3.63-9.29)^a^	5.50 (3.61-7.39)	5.94 (3.97-7.91)	4.32 (1.44-7.20)
Annual income				
<$50,000	6.74 (6.43-7.05)^a^	6.19 (5.87-6.51)	5.21 (4.92-5.50)	3.71 (3.38-4.03)
$50,000-$74,999	6.30 (5.95-6.64)^a^	5.45 (5.09-5.81)	4.58 (4.22-4.93)	3.58 (3.15-4.00)
$75,000-$100,000	6.75 (6.11-7.39)^a^	5.99 (5.41-6.56)	5.31 (4.71-5.90)	3.51 (2.88-4.14)
>$100,000	7.71 (7.02-8.40)^a^	6.59 (5.84-7.35	4.79 (4.04-5.54)	2.59 (1.80-3.38)

CI, confidence interval; VAS, Visual Analog Scale. ^a^Highest mean VAS score in a row.

Participants also rated each reconstructive flap on the categorical criteria of the overall grade, scar quality, skin color match, and skin deformity. Table 4 exhibits the odds ratios of giving the best rating in each category when evaluating an excellent dorsal flap as compared with a non-excellent dorsal flap (very good, good, or poor). The odds ratio for receiving an excellent rating for the overall grade was 4.21 (95% CI = 3.04-5.82) for excellent dorsal flap with *P* < 0.0001. The odds ratio for receiving a matte rating for scar quality was 5.20 (95% CI = 4.00-6.75) for excellent dorsal flap with *P* < 0.0001. The odds ratio for receiving a perfect rating for skin match was 4.23 (95% CI = 3.19-5.62) for excellent dorsal flap with *P* < 0.0001. The odds ratio for receiving a perfect rating for skin match was 3.16 (95% CI = 2.32-4.29) for excellent dorsal flap with *P* < 0.0001.

**Table 4.** Categorical Grading of Dorsal Rectangular and Pentagonal Flaps

	Dorsal Rectangular Flap	Dorsal Pentagonal Flap
Overall grade		
Odds ratio—getting excellent rating (95% CI)	4.21 (3.04-5.82)	1
Predicted probability—getting excellent rating	0.21	0.06
*P*-value (alpha = 0.05)	<0.0001	—
Scar quality		
Odds ratio—getting matte rating (95% CI)	5.20 (4.00-6.75)	1
Predicted probability—getting matte rating	0.81	0.45
*P*-value (alpha = 0.05)	<0.0001	—
Skin color		
Odds ratio—getting perfect rating (95% CI)	4.23 (3.19-5.62)	1
Predicted probability—getting perfect rating	0.28	0.08
*P*-value (alpha = 0.05)	<0.0001	—
Hand distortion		
Odds Ratio—getting not distorted rating (95% CI)	3.156 (2.32-4.29)	1
Predicted probability—getting not distorted rating	0.21	0.08
*P*-value (alpha = 0.05)	<0.0001	—

CI, confidence interval.

## DISCUSSION

Multiple evaluation tools have been utilized within the literature including the VAS, Vancouver Scar Scale, and Manchester Scar Scale.^5-7^ To date, no studies have attempted to provide the validation of grading aesthetic outcomes of web space reconstruction. This study demonstrates that public opinion aligns with the aesthetic evaluation of dorsal flap reconstruction of syndactyly established by Yuan et al.^7^ VAS scores were consistently and statistically significantly higher for the excellent dorsal flap than for the very good, good, and poor dorsal flaps. As expected, there was a stepwise increase in VAS scores from poor to excellent dorsal flap. However, these increases did not correlate with “perfect” VAS scores and should be utilized to understand that web space reconstruction evaluation in our sample population decreases within a smaller spectrum of the VAS scale. Thus, “excellent” outcomes have lower than expected VAS scores, and “poor” outcomes have higher than expected scores. Additionally, there were large odds ratios for receiving the best grade in overall grade, scar quality, skin color match, and skin deformity for the excellent dorsal flap as compared to the non-excellent dorsal flaps.

Aesthetic evaluation of web space reconstruction has remained limited to date with much of the previous literature focused on the technical aspects of reconstruction. Lumenta et al^6^ utilized the Vancouver Scar Scale and assessment of web creep to demonstrate favorable long-term outcomes for simple syndactyly reconstruction. However, the authors did not comment on the overall appearance of the web space reconstruction and did not apply any specific aesthetic grading tools outside of the Vancouver Scar Scale. Goldfarb et al^5^ additionally utilized the Vancouver Scar Scale as well as patient and surgeon visual analog scores to evaluate the aesthetic outcomes of web space reconstruction. The authors demonstrated that while surgeons had high-rated appearance VAS scores, patients and families reported lower VAS scores, indicating better aesthetic outcomes. This finding suggests that surgeons may be more critical than patients and families when evaluating the aesthetic outcomes.

Recently, Yuan et al^7^ utilized a modified version of the Manchester Scar Score to evaluate long-term follow-up of multiple aspects of web space reconstruction, including overall grade, description, color, matte, and distortion. Our study attempted to further this evaluation by utilizing crowdsourced opinions on Amazon MTurk in an effort to validate this aesthetic evaluation scale. Crowdsourcing has previously been demonstrated to be invaluable in assessing the public’s perception of aesthetic outcomes.^15,16^ By crowdsourcing, this study provides evidence of public perceptions of the aesthetic outcomes of dorsal reconstructive flaps in syndactyly web space reconstruction. Furthermore, this use of large sample sizes allows for validation of the modified Manchester Grading System utilized in the study of Yuan et al.^7^ These findings may allow for standardization and simplification of aesthetic outcome evaluation.

While there are many strengths to this study methodology, several limitations also exist. Inherent to many surveying methodologies is the bias that exists among individuals who electively chose to take this survey. Those with a history of syndactyly or reconstructive surgery either directly or familiar with friends and family may have been more likely to start and complete our survey. In an attempt to avoid this bias, the survey title and goals were not provided to study participants. Furthermore, the outcomes of both simple and complicated reconstructive cases were included in this survey; however, this endorses the generalizability of the grading scale utilized. Despite these potential limitations, MTurk remains a commanding tool for surveying the general US population as an indicator of patient sentiment toward surgical treatment options.

Our study is unique in that it offers validation of an aesthetic grading system for web space reconstruction. Furthermore, it is the first study to utilize crowdsourced opinions to evaluate these aesthetic outcomes in an attempt to better characterize the grading scale utilized. We found that the classification system was feasible, reliable, and valid when evaluating the aesthetic outcome outcomes of web space reconstruction. These findings provide surgeons with a readily available tool that can be completed by surgeons, patients, and family members to evaluate postoperative outcomes.

## CONCLUSIONS

This study confirms and validates the assessment of aesthetic outcomes of web space reconstruction previously investigated. By crowdsourcing survey results, our study attempts to eliminate bias and gain a broad perspective of aesthetic outcome evaluation. This evidence may guide future practice such that surgical recommendations can be made that align with the aesthetic preferences of the patient population. Future prospective studies utilizing this grading system to compare different techniques of web-space reconstruction are needed to better characterize the aesthetic outcomes.

## Supplementary Material

ojaa046_suppl_Supplementary_Figures

ojaa046_suppl_Supplementary_Appendix
