# Supplementary material for: Validating Grading of Aesthetic Outcomes of Web Space Reconstruction for Finger Syndactyly: Crowdsourcing Public Perceptions Using Amazon Mechanical Turk
Source: Aesthet Surg J Open Forum. 2020 Nov 7;3(1):ojaa046. doi: 10.1093/asjof/ojaa046 (PMC7760566; doi:10.1093/asjof/ojaa046)

**Supplemental Figures**

**Supplemental Figure 1.** Example of an “excellent” grade reconstruction using a dorsal rectangular flap with skin graft from the groin region, image has been deidentified. Image has been deidentified and reproduced with permission from Yuan et al.


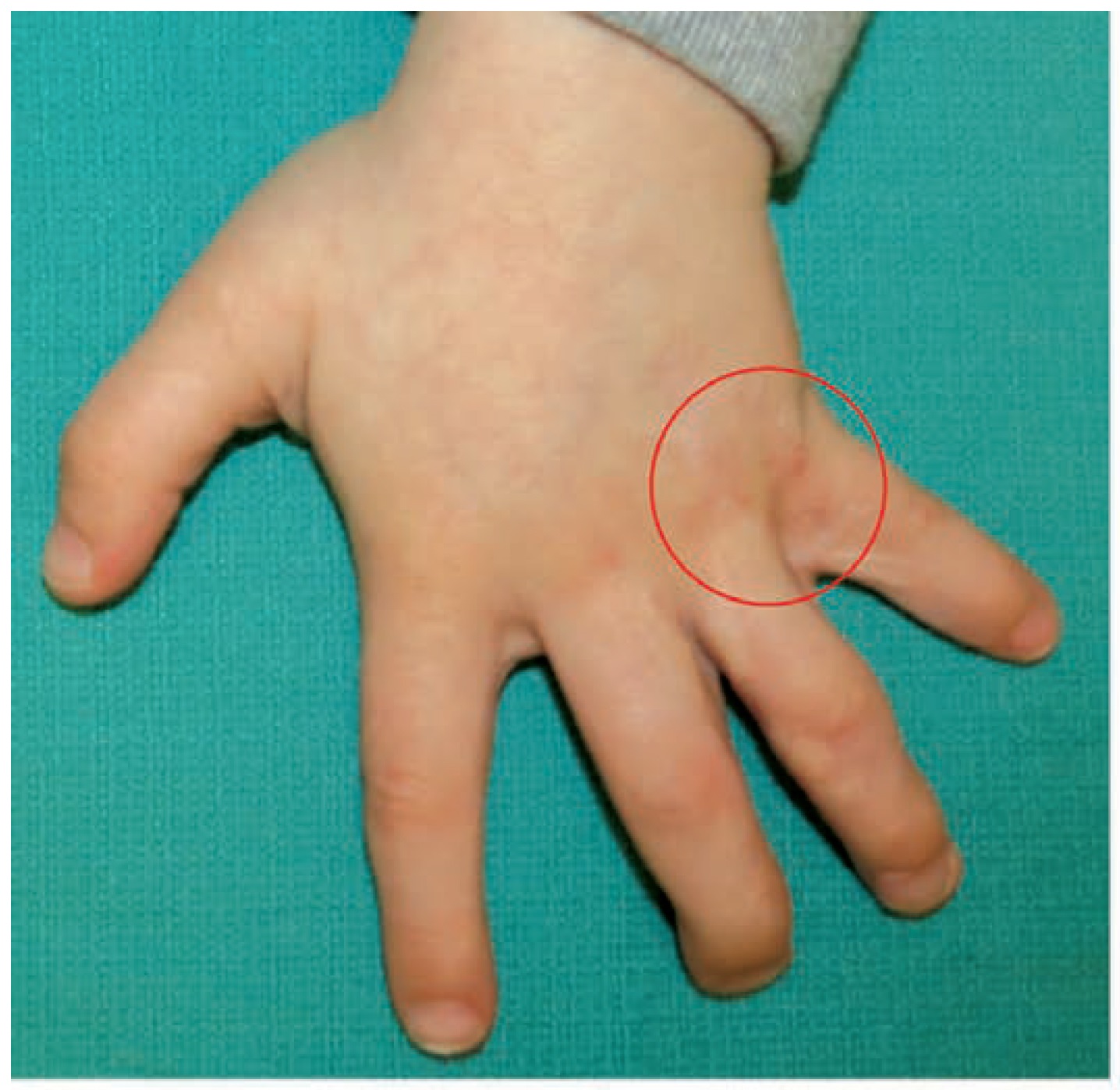


**Supplemental Figure 2.** Example of a “very good” grade reconstruction using the dorsal pentagonal advancement flap technique, image has been deidentified. Image has been deidentified and reproduced with permission from Yuan et al.


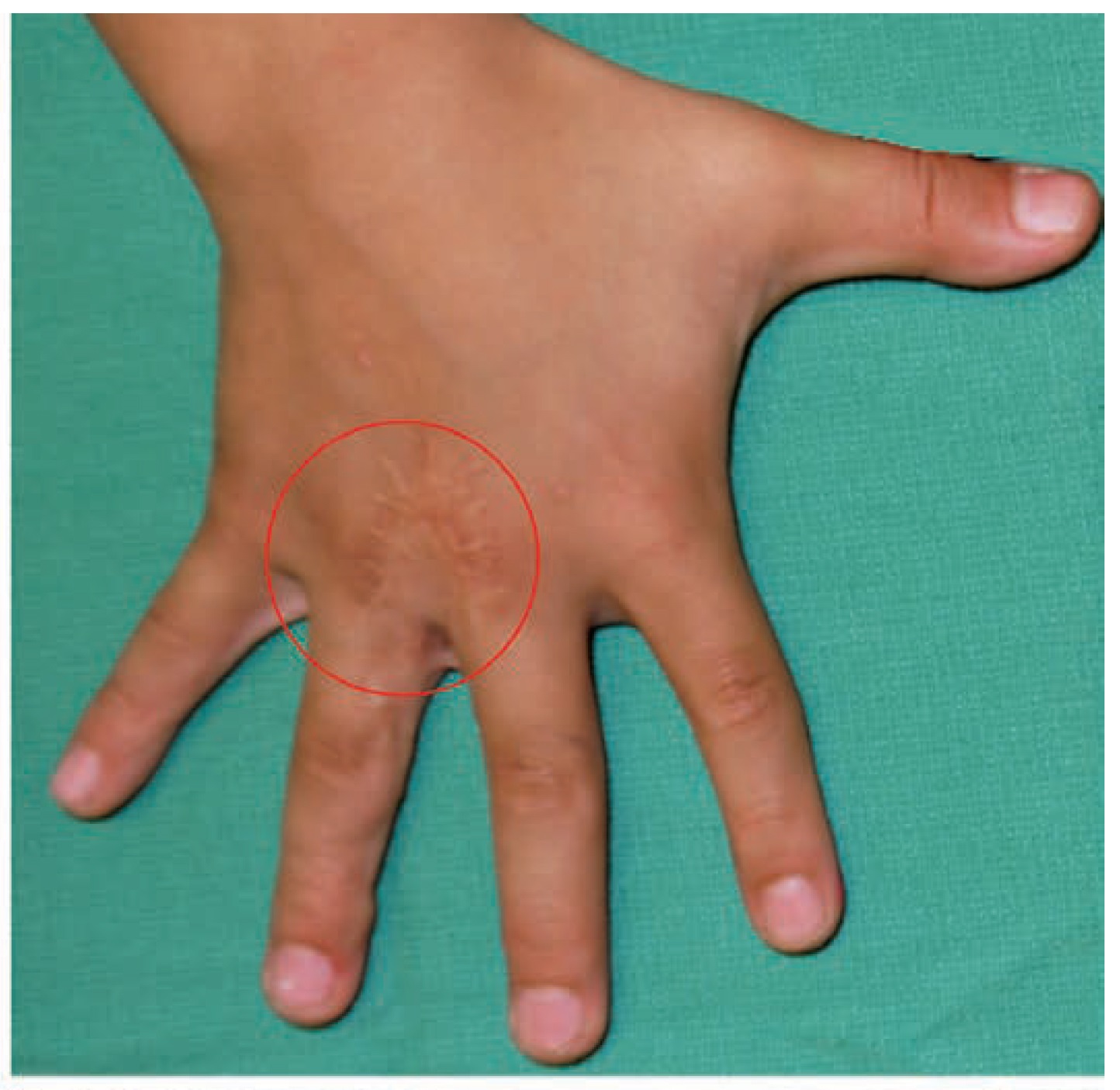


**Supplemental Figure 3.** Example of a “good” grade dorsal pentagonal advancement flap reconstruction, image has been deidentified. Image has been deidentified and reproduced with permission from Yuan et al.


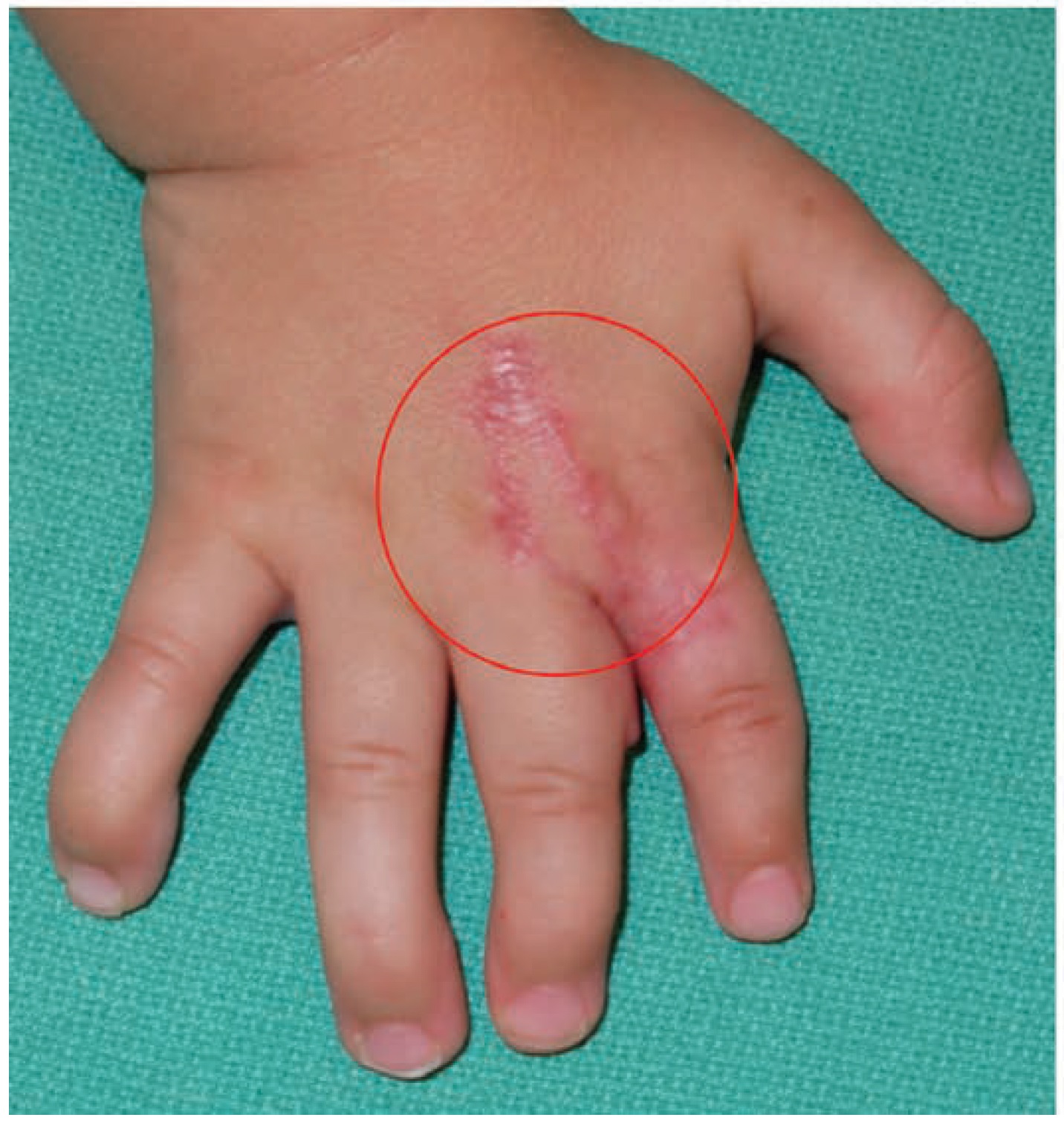


**Supplemental Figure 4.** Example of a “poor” grade dorsal pentagonal advancement flap, image has been deidentified. Image has been deidentified and reproduced with permission from Yuan et al.


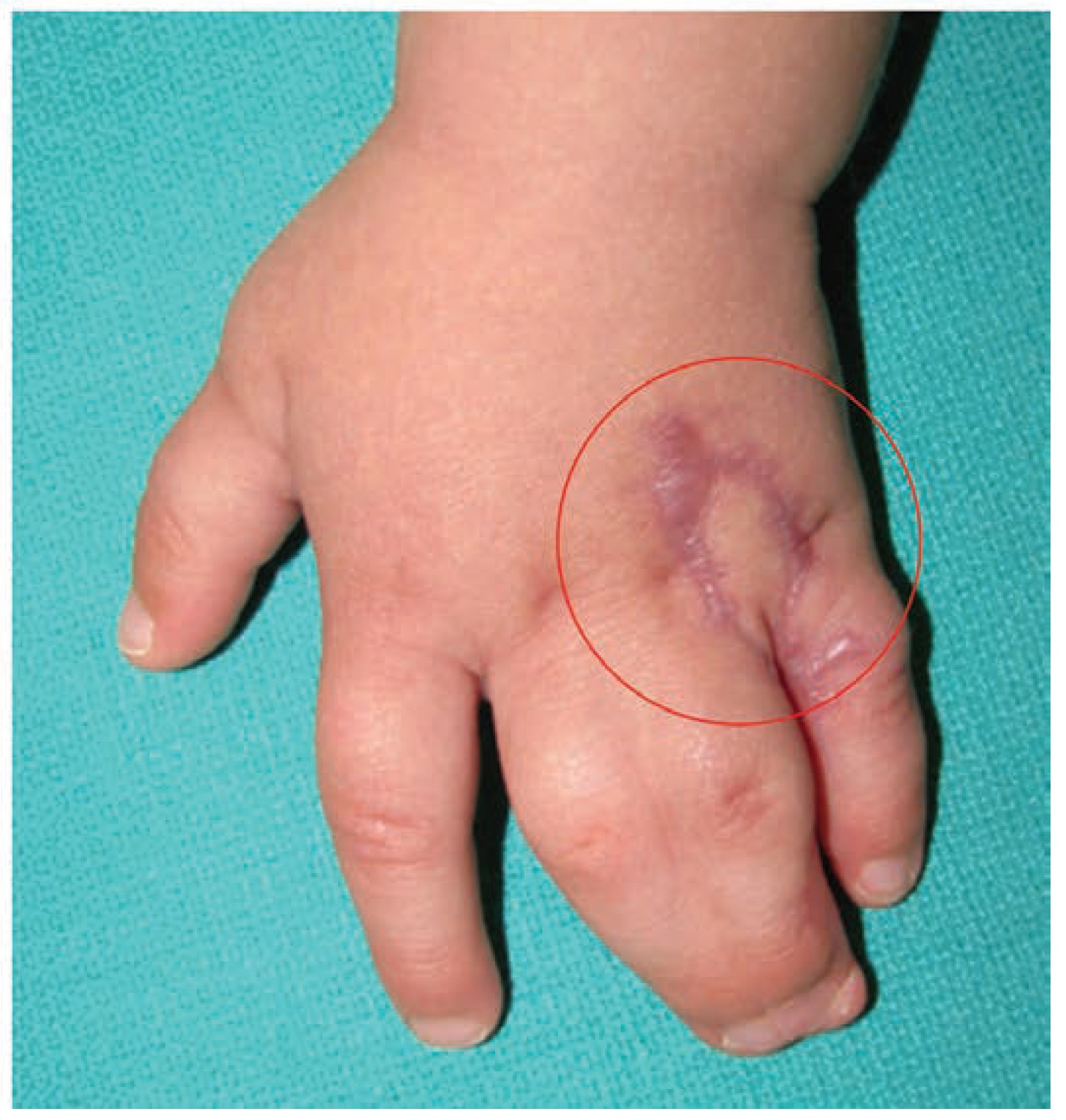

Supplement: ojaa046_suppl_Supplementary_Figures [file ojaa046_suppl_supplementary_figures.docx]
