# Supplementary material for: Validating Grading of Aesthetic Outcomes of Web Space Reconstruction for Finger Syndactyly: Crowdsourcing Public Perceptions Using Amazon Mechanical Turk
Source: Aesthet Surg J Open Forum. 2020 Nov 7;3(1):ojaa046. doi: 10.1093/asjof/ojaa046 (PMC7760566; doi:10.1093/asjof/ojaa046)
Supplement: ojaa046_suppl_Supplementary_Appendix [file ojaa046_suppl_supplementary_appendix.pdf]

Syndactyly Utility Survey

Please complete the survey below.

Thank you for your participation!

Section (1/2)

1) Please indicate your age range.
   - ◯ 18-24
   - ◯ 25-34
   - ◯ 35-44
   - ◯ 45-54
   - ◯ 55-64
   - ◯ >65

2) Please indicate your gender.
   - ◯ Male
   - ◯ Female

3) Please indicate your stated ethnicity.
   - ◯ White
   - ◯ Non-White Hispanic
   - ◯ African American
   - ◯ American Indian
   - ◯ Pacific Islander
   - ◯ Asian

4) Please indicate the approximate combined income range of your household (before taxes).
   - ◯ $100,000

Section (2/2)

Instructions: You will be presented with several images that display fingers that were reconstructed on a child's hand. After thoroughly looking at the image, please answer several questions about the appearance of the fingers and the overall appearance of the child's hand.

1. The color of the circled scar and fingers compared to the color of the child's hand.

2. Describe if the overlying skin on the scar is "matte" (not shiny) or "shiny."

3. Describe how distorted the skin looks in the reconstructed area of the hand (compared to the rest of the child's hand).

4. Provide an overall grade to the reconstructed fingers based on the criteria described in the question using two separate scales.

5) Do you understand?

◯ Yes
◯ No

Please closely look at the following reconstructed hand before answering the questions. Pay close attention to color matching, texture, and overall appearance to a normal hand/fingers.

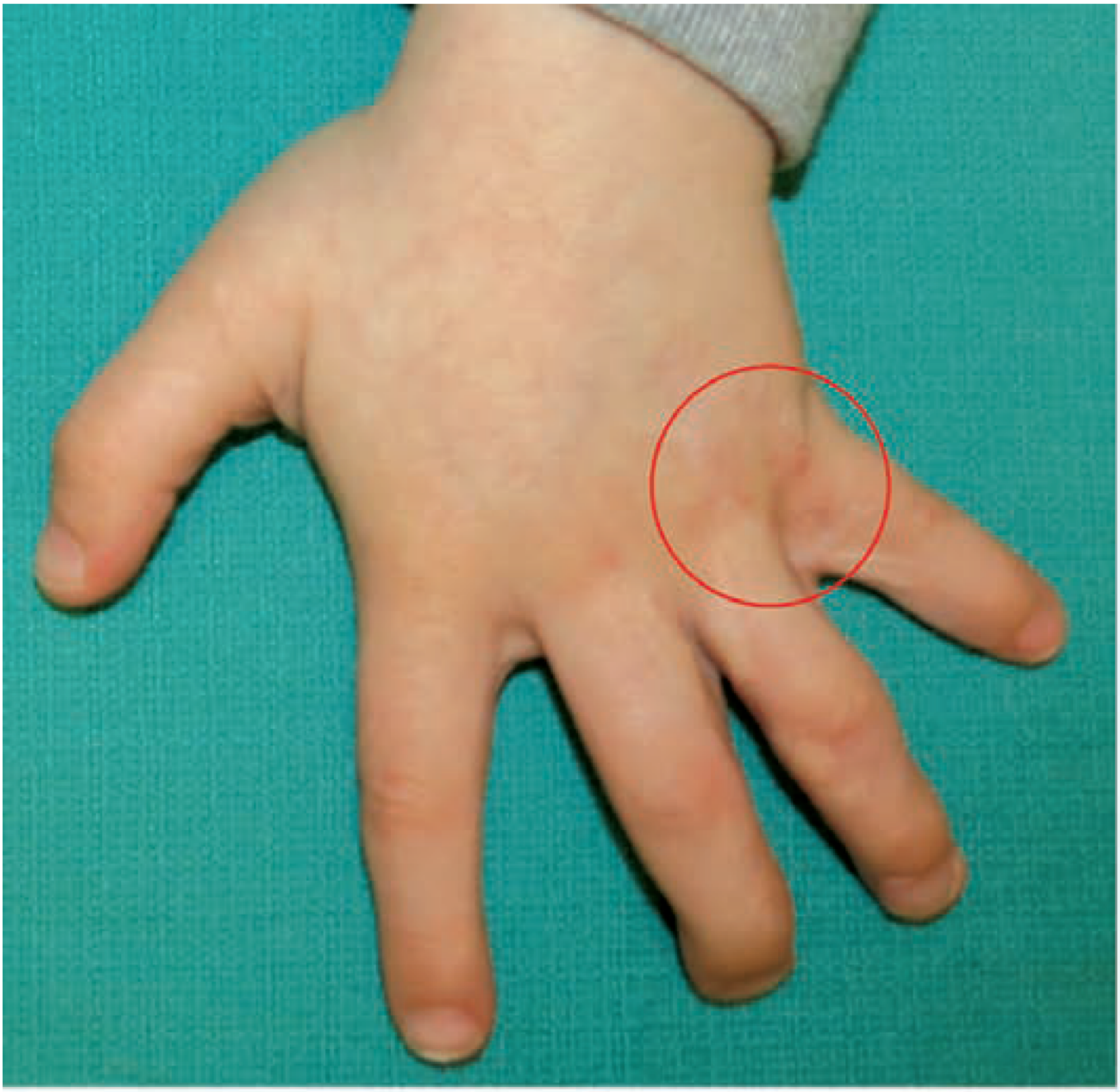

6) Please describe the skin color of the reconstructed fingers compared to the skin color of the rest of the child's hand.

- ◯ Perfect
- ◯ Slight Mismatch
- ◯ Obvious Mismatch
- ◯ Gross Mismatch

7) Please note if the skin overlying the fingers and/or scar is "matte" (not shiny) or "shiny."

- ◯ Matte
- ◯ Shiny

8) Please describe how distorted the reconstructed hand/fingers look compared to what you believe a normal hand/fingers should look like.

- ◯ Not Distorted
- ◯ Mildly Distorted
- ◯ Moderately Distorted
- ◯ Severely Distorted

9) On a scale of 0-10, where 0 represents an indistinguishable finger (does not look like a finger at all), and 10 represents a perfect looking finger/hand, how would you rate the overall appearance of this child's fingers/hand.

0 - Indistinguishable Finger — 10 - Perfect Finger

(Place a mark on the scale above)

10) Using the following criteria, and based on your answers to the above questions, please provide an overall grade to this reconstructed fingers.

Excellent = Equal appearance to surrounding skin in color, matte, and no skin distortion.

Very Good = Similar appearance to surrounding skin with mild skin distortion.

Good = Shiny appearance compared to surrounding skin with moderate skin distortion.

Poor = Obvious scare with severe skin distortion.

- ◯ Excellent
- ◯ Very Good
- ◯ Good
- ◯ Poor

Please closely look at the following reconstructed hand before answering the questions. Pay close attention to color matching, texture, and overall appearance to a normal hand/fingers.

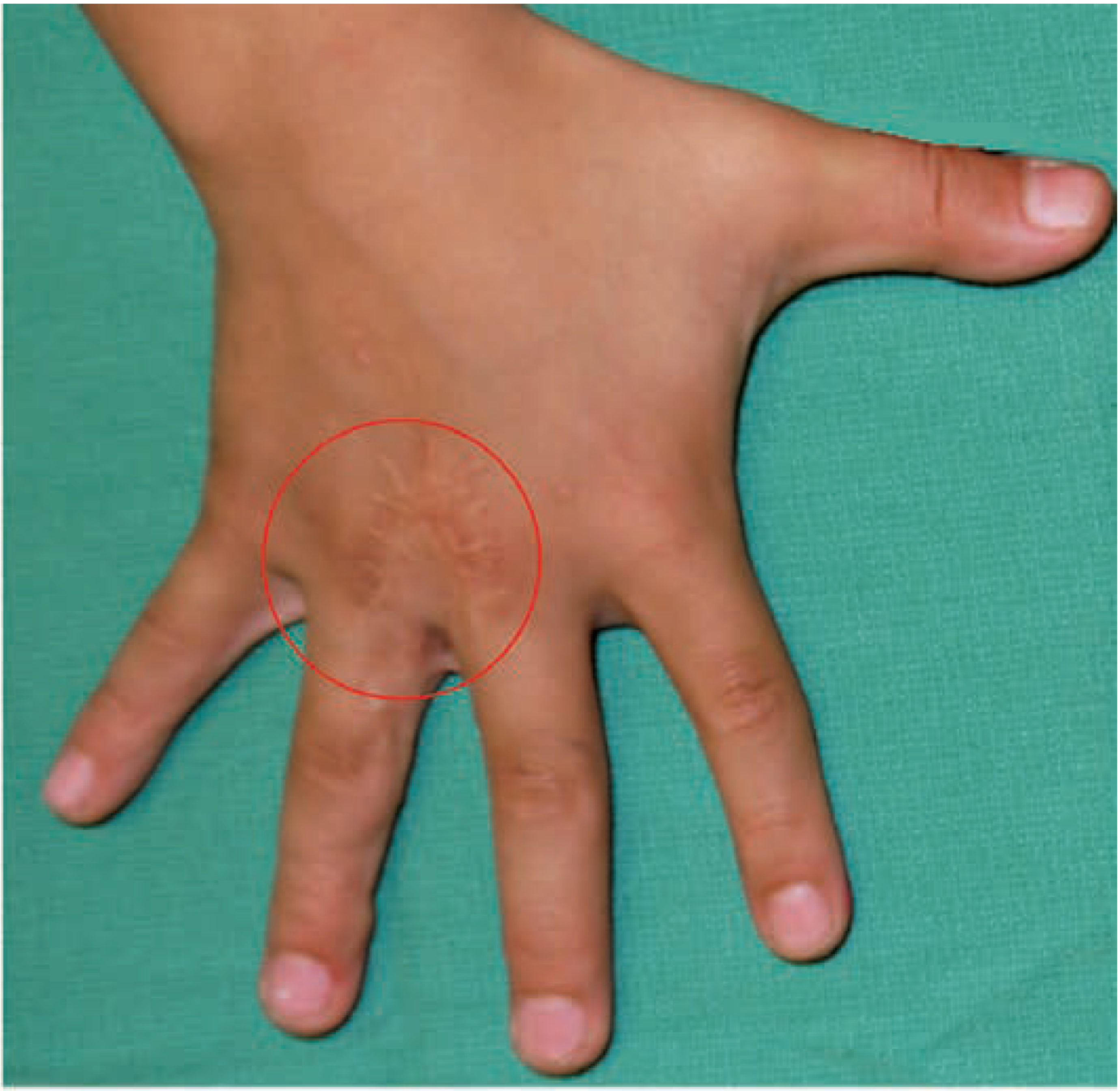

11) Please describe the skin color of the reconstructed fingers compared to the skin color of the rest of the child's hand.

- ◯ Perfect
- ◯ Slight Mismatch
- ◯ Obvious Mismatch
- ◯ Gross Mismatch

12) Please note if the skin overlying the fingers and/or scar is "matte" (not shiny) or "shiny."

- ◯ Matte
- ◯ Shiny

13) Please describe how distorted the reconstructed hand/fingers look compared to what you believe a normal hand/fingers should look like.

- ◯ Not Distorted
- ◯ Mildly Distorted
- ◯ Moderately Distorted
- ◯ Severely Distorted

14) On a scale of 0-10, where 0 represents an indistinguishable finger (does not look like a finger at all), and 10 represents a perfect looking finger/hand, how would you rate the overall appearance of this child's fingers/hand.

0 - Indistinguishable Finger | 10 - Perfect Finger

(Place a mark on the scale above)

15) Using the following criteria, and based on your answers to the above questions, please provide an overall grade to this reconstructed fingers.

Excellent = Equal appearance to surrounding skin in color, matte, and no skin distortion.

Very Good = Similar appearance to surrounding skin with mild skin distortion.

Good = Shiny appearance compared to surrounding skin with moderate skin distortion.

Poor = Obvious scare with severe skin distortion.

- ◯ Excellent
- ◯ Very Good
- ◯ Good
- ◯ Poor

Please closely look at the following reconstructed hand before answering the questions. Pay close attention to color matching, texture, and overall appearance to a normal hand/fingers.

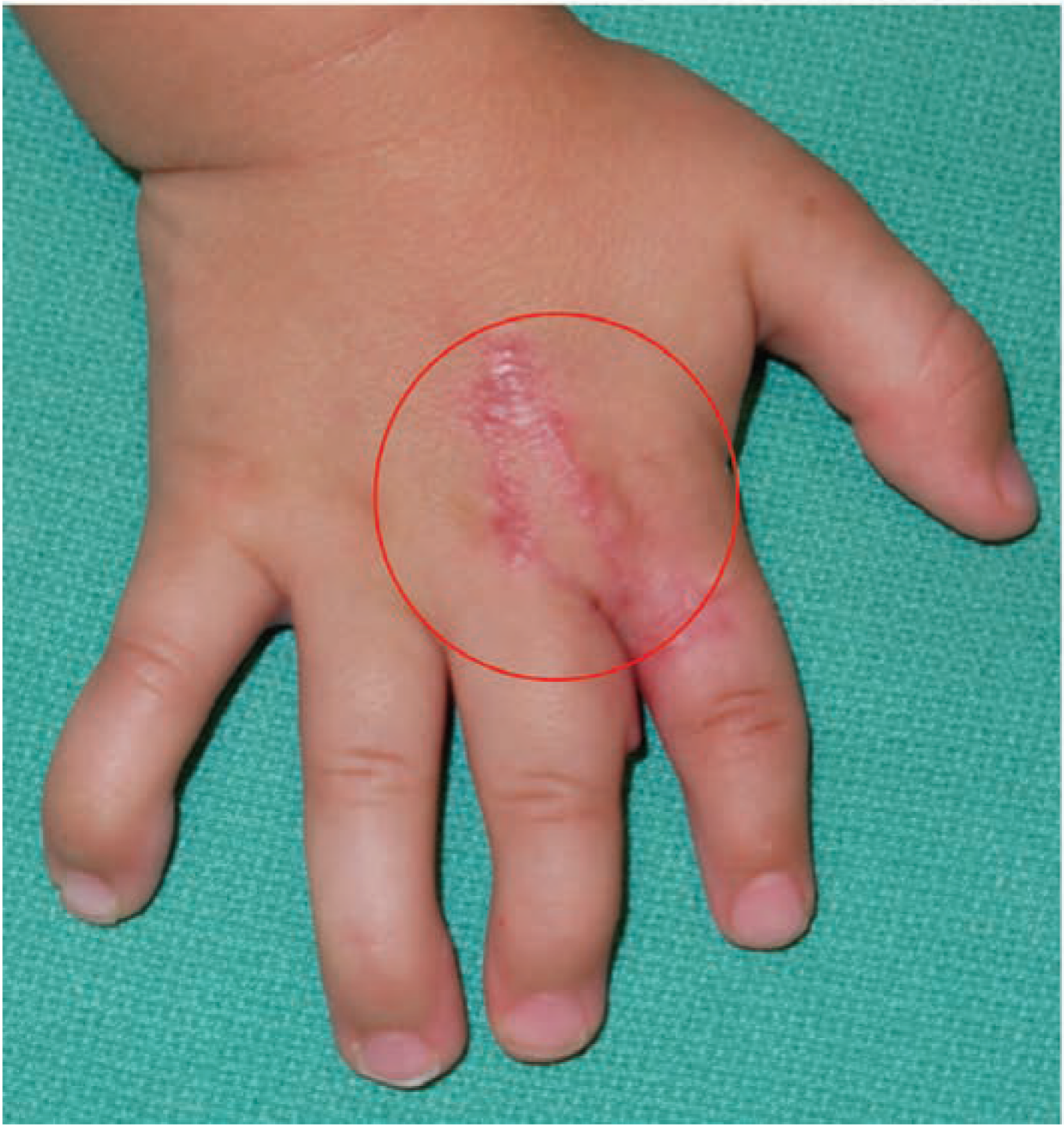

16) Please describe the skin color of the reconstructed fingers compared to the skin color of the rest of the child's hand.

- ◯ Perfect
- ◯ Slight Mismatch
- ◯ Obvious Mismatch
- ◯ Gross Mismatch

17) Please note if the skin overlying the fingers and/or scar is "matte" (not shiny) or "shiny."

- ◯ Matte
- ◯ Shiny

18) Please describe how distorted the reconstructed hand/fingers look compared to what you believe a normal hand/fingers should look like.

- ◯ Not Distorted
- ◯ Mildly Distorted
- ◯ Moderately Distorted
- ◯ Severely Distorted

19) On a scale of 0-10, where 0 represents an indistinguishable finger (does not look like a finger at all), and 10 represents a perfect looking finger/hand, how would you rate the overall appearance of this child's fingers/hand.

0 - Indistinguishable Finger | 10 - Perfect Finger

(Place a mark on the scale above)

20) Using the following criteria, and based on your answers to the above questions, please provide an overall grade to this reconstructed fingers.

Excellent = Equal appearance to surrounding skin in color, matte, and no skin distortion.

Very Good = Similar appearance to surrounding skin with mild skin distortion.

Good = Shiny appearance compared to surrounding skin with moderate skin distortion.

Poor = Obvious scare with severe skin distortion.

- ◯ Excellent
- ◯ Very Good
- ◯ Good
- ◯ Poor

Please closely look at the following reconstructed hand before answering the questions. Pay close attention to color matching, texture, and overall appearance to a normal hand/fingers.

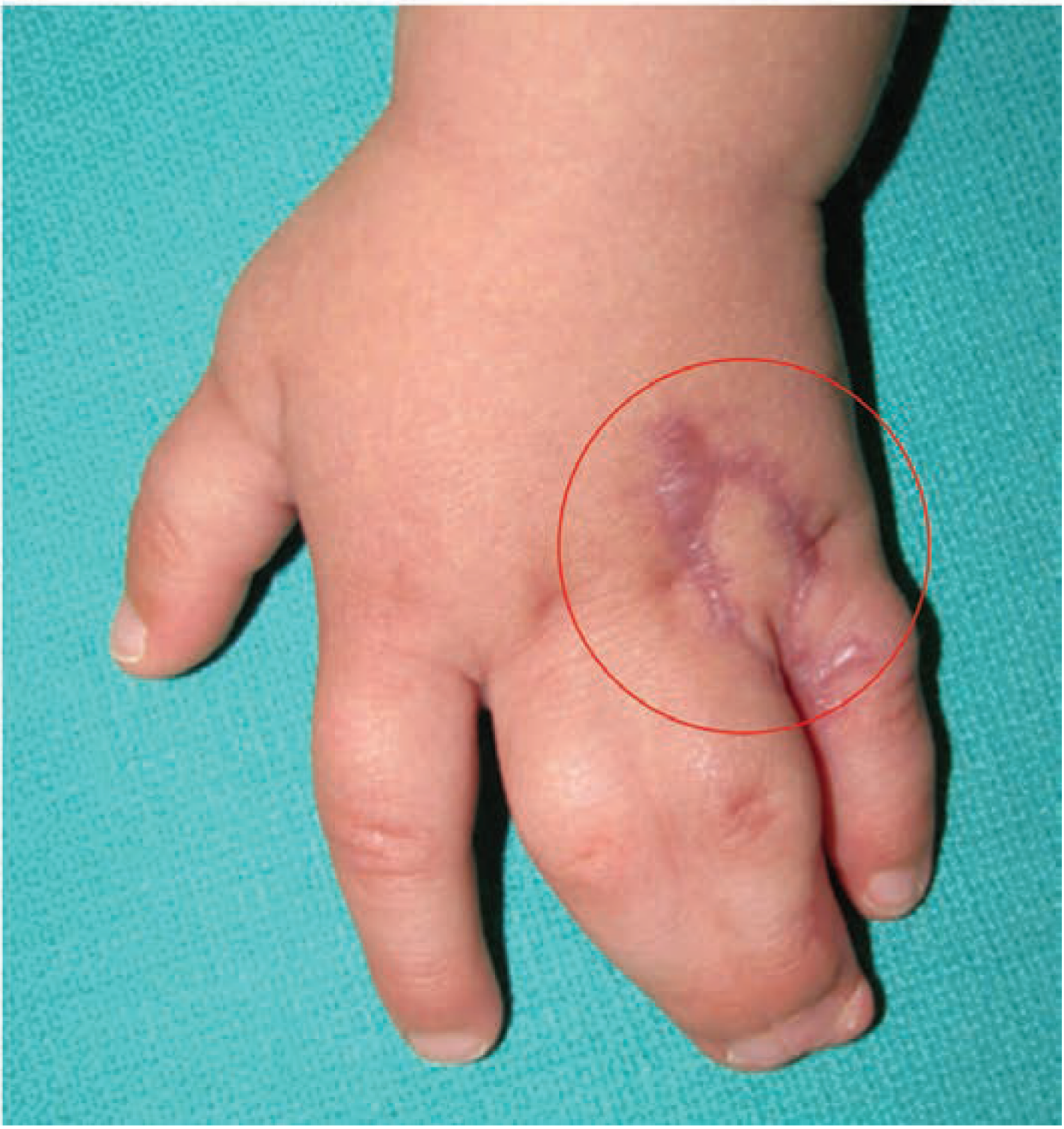

21) Please describe the skin color of the reconstructed fingers compared to the skin color of the rest of the child's hand.

- ◯ Perfect
- ◯ Slight Mismatch
- ◯ Obvious Mismatch
- ◯ Gross Mismatch

22) Please note if the skin overlying the fingers and/or scar is "matte" (not shiny) or "shiny."

- ◯ Matte
- ◯ Shiny

23) Please describe how distorted the reconstructed hand/fingers look compared to what you believe a normal hand/fingers should look like.

◯ Not Distorted
◯ Mildly Distorted
◯ Moderately Distorted
◯ Severely Distorted

24) On a scale of 0-10, where 0 represents an indistinguishable finger (does not look like a finger at all), and 10 represents a perfect looking finger/hand, how would you rate the overall appearance of this child's fingers/hand.

0 - Indistinguishable Finger — 10 - Perfect Finger

(Place a mark on the scale above)

25) Using the following criteria, and based on your answers to the above questions, please provide an overall grade to this reconstructed fingers.

Excellent = Equal appearance to surrounding skin in color, matte, and no skin distortion.

Very Good = Similar appearance to surrounding skin with mild skin distortion.

Good = Shiny appearance compared to surrounding skin with moderate skin distortion.

Poor = Obvious scare with severe skin distortion.

◯ Excellent
◯ Very Good
◯ Good
◯ Poor

Please SUBMIT the survey (press the submit button below) to receive your survey completion code.

Thank you!
